# Schädelbasismetastasen mit Hirnnervenaffektionen: Klinischer Steckbrief einer schwerwiegenden Erkrankung

**DOI:** 10.1007/s00115-021-01229-3

**Published:** 2022-01-13

**Authors:** J. Hoppe, T. Kalckreuth, M. Metelmann, J. J. Rumpf, S. Klagges, S. Dietzsch, C. Scherlach, T. Kuhnt, R. D. Kortmann, C. Seidel

**Affiliations:** 1 Department of Radiation-Oncology, University Hospital Leipzig, Leipzig, Deutschland; 2 Department of Neurology, University Hospital Leipzig, Leipzig, Deutschland; 3 Department of Neuroradiology, University Hospital Leipzig, Leipzig, Deutschland; 4 Department of Radiation-Oncology, University Hospital Freiburg, Freiburg, Deutschland; 5 Sächsisches Krebsregister Leipzig, Leipzig, Deutschland; 6 Stephanstr. 9a, 04103 Leipzig, Deutschland

**Keywords:** Schädelbasismetastasen, Hirnnervenausfälle, Stereotaktische Bestrahlung, Prostatakrebs, Brustkrebs, Skull base metastases, Cranial nerve deficits, Stereotactic radiotherapy, Prostate cancer, Breast cancer

## Abstract

**Hintergrund und Ziele:**

Schädelbasismetastasen sind eine seltene Manifestation onkologischer Erkrankungen. Wenn Hirnnerven beteiligt sind, können schon kleine Läsionen erhebliche funktionelle Beeinträchtigungen hervorrufen. Spezifische klinische Charakteristika wie neurologische Symptome, assoziierte Primärtumoren, Prognose und optimale Therapie der Erkrankung sind schlecht definiert und sollen in dieser Arbeit systematisch dargestellt werden.

**Methoden:**

Mit einem monozentrischen retrospektiven Ansatz wurden Schädelbasismetastasen bei Patienten, die im Zeitraum von 2006 bis 2018 behandelt wurden, detailliert hinsichtlich klinischer Charakteristika, der durchgeführten Therapie und des weiteren Erkrankungsverlaufs analysiert.

**Ergebnisse:**

Insgesamt 45 Patienten mit Schädelbasismetastasen und Hirnnervenausfällen wurden erfasst. Die häufigsten Primärtumoren waren Prostatakarzinom (27 %), Mammakarzinom (22 %) und multiples Myelom (16 %). Die am häufigsten betroffenen Hirnnerven waren Nervus trigeminus (42 %), Nervus oculomotorius (33 %) und Nervus facialis (27 %). 84 % aller Patienten wiesen außerhalb der Schädelbasis liegende weitere Knochenmetastasen auf. Eine durale Infiltration oder eine Meningeosis neoplastica lagen bei je 13 % der Patienten vor. Nach Bestrahlung waren 61 % der Patienten hinsichtlich der auf die Schädelbasismetastase zurückzuführenden Symptome klinisch stabil, bei 22 % hatten sich die Symptome gebessert. Das mediane Gesamtüberleben betrug 8 Monate (Spanne: 0,4–51 Monate). Bei Patienten, die mit einer dosiseskalierten Bestrahlung behandelt wurden, bestand eine längere Überlebenszeit (16,4 Monate vs. 4,7 Monate). Dieser Effekt persistierte auch in der multivariaten Analyse unter Berücksichtigung der Faktoren Karnofsky-Index, Metastasenanzahl, Primärtumor und Bestrahlungsdosis (HR 0,37, *p* = 0,02).

**Diskussion:**

Schädelbasismetastasen mit Hirnnervenausfällen haben ein vielgestaltiges Bild und oft eine schlechte Prognose. Um potenziell eine Überlebenszeitverbesserung zu erreichen, sind präzise Diagnostik und Therapie Voraussetzung. Prospektive kontrollierte Untersuchungen sind notwendig.

## Abstract (en)

**Background and purpose:**

Skull base metastases are a severe complication of various malignant tumors. If cranial nerves are involved even small lesions can cause significant symptoms. Specific clinical characteristics like neurological symptoms, associated primary tumors, prognosis and optimal treatment are poorly defined and are systematically described in this article.

**Methods:**

In a monocentric retrospective study patients with skull base metastases and cranial nerve deficits who received treatment between 2006 and 2018 were analyzed concerning clinical characteristics at initial diagnosis, treatment and course of the disease.

**Results:**

In this study 45 patients with skull base metastases and cranial nerve deficits were included. The most frequent primary tumors were prostate cancer (27%), breast cancer (22%) and multiple myeloma (16%). The most involved cranial nerves were trigeminal nerve (42%), oculomomotor nerve (33%) and facial nerve (27%). Of the patients 84% had additional bone metastases outside the skull base. Dural infiltration or meningeal carcinomatosis were each observed in 13% of the patients. After radiotherapy cranial nerve deficits remained stable in 61% of all cases and in 22% symptoms improved. Median overall survival from treatment was 8 months (range 0.4–51 months). Patients with dose-escalated radiotherapy appeared to live longer (16.4 months vs. 4.7 months). This effect persisted in a multivariate analysis including the Karnofsky index, number of metastases, primary tumor and radiation dose (HR 0.37, *p* = 0.02).

**Conclusion:**

Skull base metastases with cranial nerve deficits are complex diseases with poor prognosis. Precise diagnosis and treatment are required. Further research is needed to improve treatment.

## Hintergrund

Schädelbasismetastasen stellen eine relativ seltene und potenziell schwerwiegende onkologische Komplikation dar [2, 12]. Bis zu 4 % aller Krebspatienten weisen eine ossäre Metastasierung in die Schädelbasis auf [12, 17]. Aufgrund der hohen Komplexität dieser anatomischen Region können schon kleine Metastasen schwer beeinträchtigende Symptome hervorrufen [19, 29], besonders dann, wenn Hirnnerven beteiligt sind. Spezifische Charakteristika wie Symptome, assoziierte Primärtumoren, Prognose und optimale Therapie der Erkrankung sind jedoch wenig beschrieben. Mit der vorliegenden monozentrischen retrospektiven Analyse sollen klinische Merkmale betroffener Patienten, die erfolgte Therapie und prognostisch relevante Faktoren systematisch analysiert werden.

## Methoden

Die Krankenakten aller Patienten der Klinik für Strahlentherapie und Radioonkologie des Universitätsklinikums Leipzig zwischen 2006 und 2018 wurden systematisch durchgesehen. 45 Patienten wurden identifiziert, die mit Schädelbasismetastasen und Hirnnervendefiziten vorstellig waren. Demographische Daten, Symptome zum Zeitpunkt der Vorstellung, Verlauf der Erkrankung, Diagnostik und Therapie wurden retrospektiv erfasst. Eine Einwilligung der Patienten in die pseudonymisierte Speicherung und Auswertung ihrer Daten für wissenschaftliche Zwecke lag vor. Für die retrospektive Auswertung der Daten lag ein positives Ethikvotum der lokalen Ethikkommission vor (Nr.: 58120). Während der Therapie erfolgte mindestens einmal pro Woche eine ärztliche Untersuchung. Die Nachsorge zur Einschätzung der Remission, der neurologischen Symptome und zur Kontrolle der Therapieverträglichkeit erfolgte 2 bis 4 Wochen nach Beendigung der Behandlung. Regelmäßige onkologische Kontrolluntersuchungen erfolgten auswärtig, strahlentherapeutische Nachsorgen einmal jährlich. Sterbedaten wurden auf Basis von Informationen des sächsischen Krebsregisters bzw. nach Rücksprache mit hausärztlichen Fachkollegen ermittelt. Die Datenerfassung und -auswertung erfolgten mit SPSS 24.0. Analysen zur Erfassung prognostisch relevanter Faktoren erfolgten univariat mit Kaplan–Meier-Vergleich und Log-rank-Test sowie mittels multivariater Cox-Regression.

## Ergebnisse

Während des analysierten Zeitraums wurden 45 Patienten mit radiologisch bestätigten, durch Hirnnervensymptome auffällig gewordenen Schädelbasismetastasen erfasst. 47 % des Patientenkollektivs waren Frauen (*n* = 21) mit einem medianen Alter von 61 Jahren (Spanne: 43–78 Jahre) und 53 % Männer (*n* = 24) mit einem medianen Alter von 65 Jahren (Spanne: 30–79 Jahre). Zur radiologischen Diagnostik wurde bei 93,3 % (*n* = 42) der Patienten eine zerebrale magnetresonanztomographische (MRT-)Untersuchung durchgeführt. Eine zusätzliche computertomographische (CT-)Untersuchung erfolgte in 68,9 % (*n* = 31) der Fälle. Bei 3 Patienten erfolgte ausschließlich eine CT-Untersuchung.

**Figure 1:**
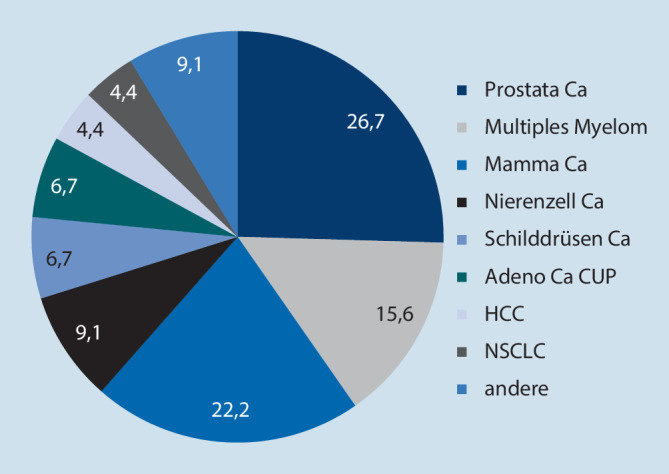

Maligne Grunderkrankungen waren Prostatakarzinom in 26,7 % (*n* = 12), Mammakarzinom in 22,2 % (*n* = 10), multiples Myelom in 15,6 % (*n* = 7), Nierenzellkarzinom in 9,1 % (*n* = 4), Schilddrüsenkarzinom und Adenokarzinom CUP in jeweils 6,7 % (jeweils *n* = 3), hepatozelluläres Karzinom und nichtkleinzelliges Lungenkarzinom (NSCLC) in jeweils 4,4 % (jeweils *n* = 2) und andere maligne Erkrankungen in 9,1 % (Ewing-Sarkom, Rektumkarzinom, Zervixkarzinom, Plattenepithelkarzinom des Oropharynx [jeweils *n* = 1]) der Fälle (Abb. 1). Bei 2 Patienten lagen mehrere maligne Tumorarten vor.

Die Zeitspanne von der Erstdiagnose des Primärtumors bis zur Vorstellung in unserer Einrichtung betrug im Median 47 Monate (Spanne 1–228 Monate). Bei 2 Patienten waren Hirnnervenausfälle die erste Manifestation der onkologischen Grunderkrankung. Hierbei handelte es sich um ein Mammakarzinom und im zweiten Fall um ein hepatozelluläres Karzinom.

Die mediane Zeitspanne von erster neurologischer Ausfallssymptomatik bis zum Beginn einer spezifischen Therapie betrug 2,7 Monate (Spanne: 0,25–24 Monate).

### Symptomatik

**Figure 2:**
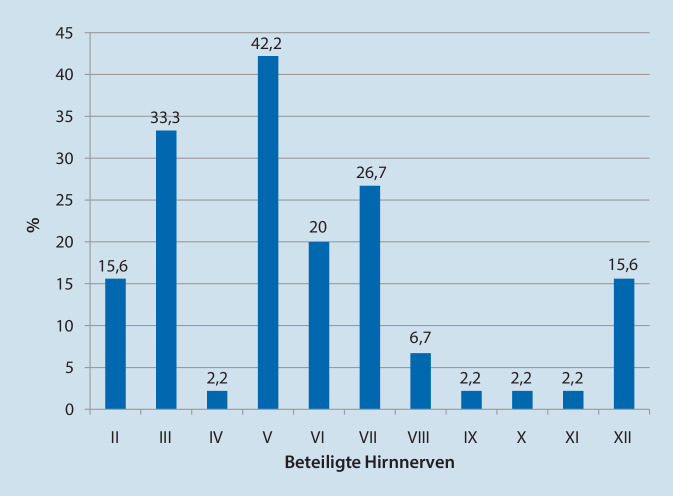

Zum Zeitpunkt der Vorstellung in unserer Einrichtung betrug der mediane Karnofsky-Index 70 % (Spanne: 40–100 %). 31,1 % (*n* = 14) der analysierten Patienten gaben bei der Vorstellung Kopfschmerzen an. Entsprechend den Einschlusskriterien hatten alle Patienten spezifische Hirnnervensymptome. Die dokumentierten Symptome betrafen folgende Hirnnerven: Nervus (N.) trigeminus (42,2 %; *n* = 19, 17/19 Hypästhesien, Parästhesien), N. oculomotorius (33,3 %; *n* = 15), N. facialis (26,7 %; *n* = 12), N. abducens (20,0 %; *n* = 9), N. opticus und N. hypoglossus (jeweils 15,6 %; *n* = 7), N. vestibulocochlearis (6,7 %; *n* = 3, 2/3 Schwindel) und N. trochlearis, N. glossopharyngeus, N. vagus, N. accessorius (jeweils 2,2 %; *n* = 1, Abb. 2).

Hirnnervenaffektionen betrafen die rechte Seite in 48,9 % (*n* = 22), die linke Seite in 31,1 % (*n* = 14) der Fälle, beidseitige Läsionen lagen bei 17,8 % (*n* = 8) der Fälle vor. Bei 42,2 % der Patienten (*n* = 19) waren mehrere Hirnnerven beteiligt. Dabei fanden sich Patienten mit 2 Hirnnervenaffektionen in 28,9 % (*n* = 13), mit 3, 4 und 5 Hirnnervendefiziten in jeweils 4,4 % (*n* = 2) der Fälle.

**Figure 3:**
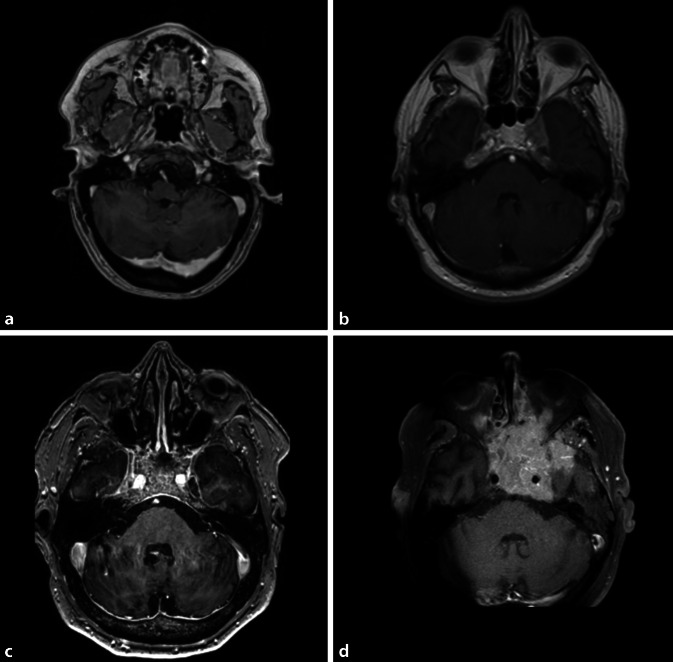

Hinsichtlich des Metastasierungsmusters zeigten 84,4 % der analysierten Patienten weitere Knochenmetastasen außerhalb der Schädelbasis, 53,3 % wiesen zusätzlich extraossäre Metastasen auf. Eine Hirnmetastasierung bestand bei 11,1 %, eine Meningeosis neoplastica oder eine durale Tumorinfiltration wurden klinisch oder radiologisch in jeweils 13,3 % (*n* = 6) der Fälle dokumentiert. Bei 69 % (*n* = 31) der Patienten waren retrospektiv kraniale MRT (cMRT-)Bilder auswertbar. Entsprechend der Einordnung nach Mitsuya [22] wurden im cMRT der Schädelbasis bei 19 % (*n* = 6/31) dieser Patienten umschriebene Läsionen, bei 39 % (*n* = 12/31) lokal infiltrierende, bei 19 % (*n* = 6/31) diffus intraossäre und bei 23 % (*n* = 7/31) diffus infiltrierende Prozesse beschrieben (Beispiele siehe Abb. 3). Übereinstimmend mit größeren Fallserien waren in Bezug auf den betroffenen Schädelbasisanteil vor allem paraselläre Anteile/Clivus und die mittlere Schädelgrube betroffen [11, 21].

### Therapie

Bei 40 der 45 Patienten wurde eine Strahlentherapie durchgeführt, die übrigen 5 Patienten erhielten aufgrund reduzierten Allgemeinzustandes keine lokale Therapie. Bei 34 Patienten erfolgte eine fraktionierte fokale Bestrahlung der Metastasenregion (27 3-D-konformale Mehrfeldertechnik, 7 intensitätsmodulierte Bestrahlung [IMRT]). Bei 4 Patienten erfolgte eine Ganzhirnbestrahlung aufgrund begleitender Hirnmetastasen, bei 2 Patienten eine stereotaktische Bestrahlung der Metastasenregion. Die Bestrahlungsenddosis betrug median 36 Gy (Spanne: 20–59,4 Gy), die Einzeldosen reichten von 1,8–5,0 Gy pro Tag. 36 der 40 Patienten beendeten die Bestrahlung nach Erreichen der geplanten Enddosis. Bei 4 der 40 Patienten musste die Bestrahlung aufgrund einer Verschlechterung des Allgemeinzustandes vorzeitig abgebrochen werden.

### Outcome und Überleben

Von 34 Patienten, die eine Strahlentherapie erhielten, waren Überlebensdaten verfügbar. Das mediane Gesamtüberleben nach Beginn der Bestrahlung betrug 7,6 Monate (Spanne: 0,06–51 Monate). Bezogen auf die drei häufigsten Primärtumoren Prostatakarzinom, Mammakarzinom und multiples Myelom betrug das Gesamtüberleben im Median 7,2 Monate (Spanne: 0,06–17 Monate), 6,5 Monate (Spanne: 0,6–22,4 Monate), 1,6 Monate (Spanne: 0,3–3,6 Monate), (*p* = 0,33). Eine Korrelation von Zeitspanne zwischen Beginn der Symptomatik und Beginn der Strahlentherapie zur Überlebenszeit bestand nicht (*p* = 0,97).

Von den 36 Patienten, die die Radiotherapie abschlossen, war die neurologische Symptomatik bei der Mehrheit der Patienten während der Therapie stabil (61 %, *n* = 22). Bei 22,2 % der Patienten (*n* = 8) wurde eine Verbesserung der neurologischen Defizite, bei 16,7 % (*n* = 6) eine Verschlechterung des Zustandes dokumentiert.

**Figure 4:**
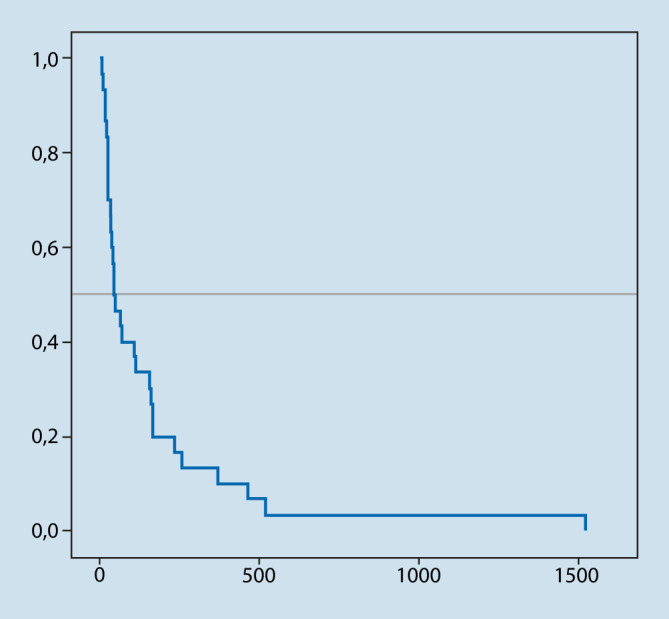

Bei Patienten (*n* = 9), bei denen Gesamtstrahlendosierungen von ≥ 45 Gy verwendet wurden, lag die mittlere Überlebenszeit bei 16,4 Monaten (Median 15,4 Monate), während Patienten (*n* = 25) mit geringeren Bestrahlungsdosierungen im Mittel 4,7 Monate (Median 1,7 Monate) lang lebten (log-rank, *p* = 0,01, Abb. 4). Für die Faktoren Primärtumor (*p* = 0,54), Karnofsky-Index (KPI) (*p* = 0,12) und Metastasenanzahl (1, 2–4, > 4), (*p* = 0,63) war kein signifikanter Effekt nachweisbar.

### Multivariate Analyse

In einer multivariaten Cox-Regressionsanalyse der Faktoren KPI, Metastasenanzahl, Primärtumor und Bestrahlungsdosis zeigten die Faktoren Bestrahlungsdosis (HR 0,37, *p* = 0,02, KI 0,16–0,86) und Karnofsky-Index einen signifikanten Effekt (HR 0,97, *p* = 0,03, KI = 0,94–0,99). Für die Anzahl der Metastasen (*p* = 0,34) und die Art des Primärtumors (*p* = 0,37) war kein signifikanter Effekt nachweisbar.

## Diskussion

Schädelbasismetastasen mit Hirnnervendefiziten traten in unserer Analyse überwiegend als Metastasen von Prostatakarzinom, Mammakarzinom oder multiplem Myelom auf. In der Literatur dominieren Fallbeschreibungen von Patienten mit Prostatakarzinom [19, 27, 28, 30] und multiplem Myelom [8, 22–24]. Beschreibungen von Patientinnen mit Mammakarzinom sind etwas seltener zu finden [5, 6, 18, 19]. Entsprechend des betroffenen anatomischen Kompartiments sind Schädelbasismetastasen weniger mit Hirnmetastasen, als vielmehr mit anderen Knochenmetastasen assoziiert. Überwiegend zeigten die Hirnnerven III bis VII Ausfallssymptome und die paraselläre Region bzw. Region der mittleren Schädelgrube erschienen, übereinstimmend mit den Ergebnissen anderer Autoren (Tab. 1), vermehrt betroffen. Über Gründe hierfür (z. B. unterschiedliche Länge der intraossären Nervenverläufe, Knochenvaskularisationsunterschiede) kann teilweise nur spekuliert werden. Im Fall des N. trigeminus ist es möglich, dass neben der Länge des Verlaufs mit drei Ästen in der Schädelbasis auch eine überzufällig häufige Affektion des Ganglion Gasseri eine Rolle spielt [2]. Schädigungen der kaudalen Hirnnerven sind möglicherweise klinisch unterdiagnostiziert, nur selten erfolgte initial eine dezidierte Schluckdiagnostik. In mehr als 25 % aller Fälle war bildmorphologisch eine zusätzliche durale Infiltration oder eine Meningeosis neoplastica auffällig, die aufgrund ihrer Implikationen für das Therapiekonzept nicht übersehen werden sollten. Im Falle ausgedehnter duraler Infiltration müssten entsprechend die Zielregionen der Bestrahlung deutlich erweitert werden. Bei bildmorphologischem Nachweis einer Meningeosis neoplastica muss ergänzend eine Lumbalpunktion erfolgen und eine Änderung systemischer Therapie bzw. Hinzunahme intrathekaler Therapie geprüft werden.

**Table 1:** 

Syndrom	Dominierende Hirnnervendefizite	Diese Serie	Mitsuya et al. (2011) [21]	Greenberg et al. (1981) [11]
Orbitales Syndrom	II–IV	3 (7 %)	4 (20 %)	3 (7 %)
Paraselläres Syndrom	III–VI	16 (36 %)	7 (35 %)	7 (16 %)
Syndrom der mittleren Schädelgrube	V, VII	18 (40 %)	4 (20 %)	15 (35 %)
Foramen-jugulare-Syndrom	IX, X, XII	3 (7 %)	3 (15 %)	9 (21 %)
Syndrom der okzipitalen Kondyle	XII	–	2 (10 %)	9 (21 %)
Mehrere Schädelbasisbereiche	Multiple	5 (11 %)	NA	NA
Patienten (gesamt)	–	45	20	43

*NA* Not available

Von den ersten neurologischen Symptomen bis zur Therapie vergingen im Mittel knapp 3 Monate. In frühen publizierten Serien war eine Symptomverbesserung bei bis zu 87 % der Fälle festzustellen, wenn Symptome weniger als 1 Monat bestanden, hingegen remittierten klinisch nur 25 % der Patienten im Falle einer Symptompersistenz > 3 Monate [11, 29]. Die Patienten der untersuchten Kohorte gehörten somit a priori einer Gruppe mit einer entsprechend schlechteren Prognose hinsichtlich einer Symptomkontrolle an.

Entsprechend dieser zeitlichen Dringlichkeit sollte nach Diagnose von Hirnnervendefiziten nach „unauffälliger zerebraler“ Bildgebung keinesfalls selbstverständlich ein „idiopathischer“ Nervenausfall diagnostiziert werden. Möglichst sollte eine dünnschichtige MRT der Schädelbasis mit T1 ± Kontrastmittel und T2-Sequenzen [10, 21] und eine Lumbalpunktion Teil der Differenzialdiagnostik bei unklaren Hirnnervendefiziten, insbesondere bei Malignomen in der Vorgeschichte, sein. Bildmorphologisch imponieren Schädelbasismetastasen in der T1-Sequenz verdrängend auf das normale T1-hyperintense Fettsignal und erscheinen eher hypointens bei variablem T2-Signal [16]. Kleine Metastasen können teilweise nur subtile Veränderungen hervorrufen. Nach Mitsuya et al. können umschriebene, umschrieben infiltrative, diffus osteolytische und diffus infiltrative Schädelbasismetastasen unterschieden werden ([21]; Abb. 3).

Ergänzende empfohlene diagnostische Methoden sind eine dünnschichtige kraniale Computertomographie (CCT) und eine Knochenszintigraphie, deren Sensitivität teilweise allerdings für osteolytische Läsionen eingeschränkt ist [13], und eine Positronenemissionstomographie-CT (PET-CT; [9]), deren Auflösung im Bereich der Schädelbasis in Anbetracht der Glukoseaufnahme des Hirns jedoch ebenfalls limitiert ist. Beim Prostatakarzinom bietet ein prostataspezifisches Membran-Antigen-PET-CT (PSMA-PET-CT) eine hohe Sensitivität [1, 14].

Die mediane Überlebenszeit der Patienten war trotz Therapie mit 8 Monaten kurz. Überwiegend scheinen Schädelbasismetastasen Ausdruck eines multipel metastasierten Spätstadiums der zugrunde liegenden Tumorerkrankung mit schlechter Gesamtprognose zu sein [7, 15, 25]. Zu den Todesursachen gibt es aus den veröffentlichten Serien und auch aus unserer Kohorte leider keine exakten Daten. In Anbetracht hoher klinischer Ansprechraten nach lokaler Strahlentherapie wie bei Dröge et al. (10 % Komplettremission, 70 % partielle Remission; [7]) und McDermott et al. (66 % Komplettremission, 27 % partielle Remission; [19]) und auch in unserer Serie (80 % Remission oder Stabilisierung) liegt als Haupttodesursache ein Tod durch systemische Progression der Grunderkrankung näher. Diese Annahme lässt sich aber in Ermangelung einer standardisierten Nachsorge, der Kenntnis der Remissionsdauer und der bildgebenden Kontrollen von Schädelbasis und Ganzkörper nicht belegen.

In einigen Serien fällt auf, dass moderat hypofraktionierte Bestrahlungskonzepte der Schädelbasis (z. B. 3,0–30,0 Gy, biologisch äquivalente Dosis 39 Gy) mit sehr schlechter Prognose und Überlebenszeiten um 3 Monate im Median verbunden waren [7, 25, 29], während hochdosierte Konzepte mit Einzeitbestrahlungen (z. B. 16 Gy Einzeldosis, biologisch äquivalente Dosis 42,6 Gy) mit einem besseren medianen Überleben assoziiert schienen (7–15 Monate; [4, 15, 26]; Tab. 2).

**Table 2:** 

	Jahreszahl	Patientenzahl	Bestrahlungskonzept	Lokale Ansprechrate	Medianes Überleben
Greenberg et al. [11]	1979	43	Strahlentherapie, zur Dosierung k. A.	Wenn Symptome < 1 Monat 72 % (klinisch), wenn Symptome > 1 Monat 28 % (klinisch)	k. A.
Vikram et al. [29]	1979	46	Strahlentherapie, fraktioniert: ED 3,0 Gy, GD 30,0 Gy	Wenn Symptome < 1 Monat 87 % (klinisch), wenn Symptome 1–3 Monate 69 % (klinisch), wenn Symptome > 3 Monate 25 % (klinisch)	*Mammakarzinom/Kopf-Hals-Tumoren*:
					10 Monate
					*Lymphom/Lungenkarzinom*
					3 Monate
Dröge et al. [7]	2013	30	3‑D-konformale, fraktionierte Strahlentherapie: ED 3,0 Gy, GD 30,0 Gy	70 % PR, 10 % CR	3,9 Monate
O’Sullivan et al. [25]	2003	32	3‑D-konformale, fraktionierte Strahlentherapie, 90 % der Patienten: ED 4,0 Gy, GD 20,0 Gy	25 % PR, 25 % CR	3 Monate
Iwai et al. [15]	1998	18	Gamma-Knife Radiochirurgie, Einzeit GD 16,2 Gy (12–23 Gy)	61 % PR	7,4 Monate
Coppa et al. [4]	2009	31	Cyber-Knife Radiochirurgie, fraktioniert: GD 12,5–35 Gy	74 % PR + SD	9,7 Monate
Pan et al. [26]	2011	27	Gamma-Knife Radiochirurgie, Einzeit GD 14 Gy (10–16 Gy)	44 % PR	15 Monate

*ED* Einzeldosis, *GD* Gesamtdosis, *PR* „partial remission“, *CR* „complete remission“, *SD* „stable disease, *k.a.* keine Angabe“

Auch in unserer Serie erschien eine lokal hochdosierte Bestrahlung in der univariaten und multivariaten Analyse unter Berücksichtigung anderer prognostisch relevanter Faktoren (Karnofsky-Index, Anzahl der bekannten Metastasenregionen) mit einem Überlebensvorteil assoziiert. Allerdings kann eine Überlegenheit der hochdosierten Bestrahlung mit unseren Daten nicht bewiesen werden, da ein Effekt anderer Faktoren (z. B. ein Selektionseffekt auf Basis der Größe des bestrahlten Zielvolumens) nicht gemessen wurde und nicht ausgeschlossen werden kann. Zudem verstarben einige Patienten bereits früh während der Therapie und führen somit zu einer Imbalance der Gruppen zuungunsten der Prognose in der Niedrigdosisgruppe. Insgesamt sind die Daten in Anbetracht ihrer retrospektiven Erhebung und ohne standardisierte bildmorphologische Nachsorge mit Vorsicht zu interpretieren. Wir können nur spekulieren, dass eine suffiziente lokale Bestrahlung eine Verschlechterung vital relevanter Symptome (z. B. einer Schluckstörung mit Pneumonieneigung) mittelfristig vermeiden kann und dass zudem posttherapeutisch gebesserte neurologische Defizite eine weitere tumorspezifische Systemtherapie ermöglichen.

Bei kleinen, gut abgrenzbaren Volumina erscheint es plausibel, eine stereotaktische Bestrahlung bzw. Radiochirurgie zu verwenden [2, 4, 26]. Chirurgische Verfahren [20] kommen wegen des Risikos für posttherapeutische neurologische Defizite nur sehr selten in Betracht. Komplikationen der stereotaktischen Strahlentherapie der Schädelbasis sind hingegen selten und variieren zwischen 6 und 8,5 % [3, 15]. Ob bei größeren Zielvolumina eine fraktionierte Bestrahlung mit moderat erhöhter Enddosis z. B. von 2,5–45,0 Gy einen Vorteil bringt, erscheint nach unseren Daten offen. Leider existiert kein etabliertes standardisiertes Zielvolumenkonzept bei der Bestrahlung der Schädelbasis, entsprechend sollten pragmatisch MR-morphologisch befallene Regionen der Schädelbasis mit Schwerpunkt auf die Bereiche der betroffenen Hirnnerven und die benachbarte Dura sowie ein anatomisch angepasster Sicherheitssaum (z. B. von 2 cm) in das klinische Zielgebiet eingeschlossen werden. Bei schlecht abgrenzbaren Läsionen kann pragmatisch die gesamte knöcherne Schädelbasis von den Keilbeinflügeln beidseits über den Clivus und die medialen Anteile des Os temporale und die angrenzende Dura eingeschlossen werden. Dosisbeschränkungen („dose constraints“) für den optischen Apparat, die Innenohren und den Hirnstamm sollten beachtet werden.

## Schlussfolgerung

Eine ossäre Metastasierung in die Schädelbasis sollte insbesondere bei bekanntem Prostatakarzinom, Mammakarzinom oder multiplem Myelom und Hirnnervenausfällen bedacht werden. Bereits frühzeitig ist eine entsprechende Diagnostik, insbesondere ein dünnschichtiges Schädelbasis-MRT indiziert. Durale Infiltrationen oder Meningeosis neoplastica sollten nicht übersehen werden. Zur Symptomverbesserung sind eine gesicherte Diagnose und aus den Ergebnissen der Literatur ein zeitnaher Therapiebeginn wichtige Basis. Eine Strahlentherapie kann dann in den meisten Fällen mindestens zu einer Symptomstabilisierung führen. Bei kleinen Zielvolumina erscheint die Anwendung hochdosierter Präzisionstechniken (Einzeitstereotaxie/Radiochirurgie) und bei schwer abgrenzbaren/diffusen Prozessen ein konventionelles Konzept, evtl. mit moderat erhöhter Enddosis (z. B. 2,5–45,0 Gy) vertretbar. Prospektive, multizentrisch kontrollierte Studien erscheinen spezifisch für Schädelbasismetastasen wünschenswert, um Evidenz höherer Qualität zu generieren.

## Fazit für die Praxis

Schädelbasismetastasen sollten als Ursache von Hirnnervenausfällen insbesondere bei Patienten mit bekannten Tumoren in Betracht gezogen und genau diagnostiziert werden. Eine lokale Strahlentherapie kann die neurologische Ausfallsymptomatik und die Prognose dieser schwerwiegenden Erkrankung verbessern. Bessere Evidenz erscheint für eine Optimierung der Behandlung nötig.
